# Parental COVID-19 vaccination hesitancy among parents of children aged 5–18 years in Thailand: a cross-sectional survey study

**DOI:** 10.1186/s40545-022-00455-7

**Published:** 2022-10-06

**Authors:** Pantira Parinyarux, Kanokkarn Sunkonkit, Kitiyot Yotsombut

**Affiliations:** 1 Department of Social Pharmacy, Faculty of Pharmacy, Payap University, Chiang Mai, Thailand; 2 Division of Pulmonary and Critical Care, Department of Pediatrics, Faculty of Medicine, Chiang Mai University, Chiang Mai, Thailand; 3 Department of Pharmacy Practice, Faculty of Pharmaceutical Sciences, Chulalongkorn University, Bangkok, 10330 Thailand

**Keywords:** COVID-19, Vaccination hesitancy, Vaccination refusal, Parents, Children

## Abstract

**Background:**

To promote an acceptance rate of COVID-19 immunization among Thai children, concerns about parental vaccination hesitancy should be urgently studied. This study aimed to examine the parental COVID-19 vaccination hesitancy (PVh) level and influencing factors among Thai parents of children 5–18 years of age.

**Methods:**

This cross-sectional survey was conducted in Thailand during May and June of 2022. The Google forms for data collection were distributed to parents (a father, a mother, or one who nurtures and raises a child) via various online social media. Data regarding PVh level, relevant attitudes, experiences of COVID-19 and COVID-19 vaccination (EC&V), and family contexts (FC) were collected and analyzed using descriptive statistics. Mann–Whitney *U* test was used to compare the differences among groups of parents based on EC&V and FC. The factors influencing PVh were assessed by multiple regression analysis.

**Results:**

Four hundred and eighty-eight parents completed the online questionnaire. Their median (IQR) age was 41 (35–47) years. They lived in different provinces from all regions across Thailand. Ninety percent of them were authorized persons to make decision about children vaccination. Fifty-eight percent of the respondents had vaccine hesitancy, defined as PVh level at moderate or above. Parents who had ever refused COVID-19 vaccination for themselves or refused to vaccinate their children against any other diseases had statistically significant higher levels of PVh (*p* value < 0.001). Conversely, the parents who had finished the initial COVID-19 vaccine had lower PVh levels with statistical significance (*p* value = 0.001). Attitude towards COVID-19 (AC), attitude towards COVID-19 vaccine (AV), and perceived behavioral control (PC) of the parents negatively influenced PVh with statistical significance, according to the results of the multiple regression analysis (Betas = − 0.307, − 0.123, and − 0.232, respectively).

**Conclusions:**

COVID-19 vaccination hesitancy was commonly found among Thai parents. The factors of the hesitancy were multifaceted. Different experiences regarding COVID-19 vaccination for themselves and any vaccinations for their children were associated with different PVhs. The attitudes especially AC, AV, and PC statistically influenced PVh. These findings should be exploited for national and local policy planning as well as public campaigns.

## Background

The coronavirus disease 2019 (COVID-19) is an emerging contagious disease caused by the SARS-CoV-2 that was recognized by the World Health Organization (WHO) as emerging global health on January 30, 2020, due to its rapid spread to all other countries around the globe [1, 2]. Although various preventive measures including social distancing, good hand hygiene with alcohol rubbing, as well as mask-wearing have been advocated, the number of COVID-19-infected persons is still rising. Due to the infectious nature of the disease, immunization was hopefully expected to be one of the most effective ways to fight the COVID-19. As such, the COVID-19 pandemic situation fostered the development of vaccines against the disease with various newly invented platforms [3]. The vaccination program in Thailand has been implemented since February 28, 2021 [4]. The overall rate of completed initial COVID-19 vaccination among Thai people is around 76%, mainly adults and elderly. Besides, only 54.1% of Thai children have received the complete initial COVID-19 vaccination [5]. Based on the estimated R_0_ of COVID-19 ranged from 1.4 to 6.68, the herd immunity threshold would range from 28.57 to 85.03% [6]. The available COVID-19 vaccines are effective in reducing morbidity and mortality, rather than infection prevention. Therefore, at least 85% of Thai people should receive the complete course of COVID-19 vaccine. These data pointed out that there was an urgent need to promote access to vaccination programs for both adults and children in Thailand.

Although the perceived severity of COVID-19 in children is less than in adults, long-term serious complications of COVID-19 in children have been increasingly reported including long-COVID symptoms and multisystem inflammatory syndrome in children (MIS-C). These complications could be prevented by COVID-19 vaccines. As a result, every child should be managed to receive the vaccine timely [7]. On January 5, 2022, the Food and Drug Administration (FDA) of Thailand approved the Pfizer BioNTech mRNA COVID-19 vaccine for Thai children aged 5 years and older [8, 9]. However, the rate of vaccine acceptance among children aged 5–11 years and 12 years and older in Thailand is still lower than the recommended herd immunity threshold.

Vaccine hesitancy has long been one of the major obstacles to immunization among people of all age groups. The WHO defined vaccine hesitancy as “a delay in acceptance or refusal of vaccination even though vaccination services are available”. Factors that determine individual vaccine hesitancy consists of (1) complacency which indicates a low perceived risk of disease (2) confidence in the safety and efficacy of the vaccine (3) convenience in acquiring and accessing vaccines [10–12]. In the case of COVID-19 vaccination, studies have found that the main causes of vaccine hesitancy were concerns about the safety and potential side effects and distrust in the vaccine efficacy and quality. Given that COVID-19 vaccines were manufactured by brand-new production platforms without long-term safety evaluation, misinformation regarding COVID-19 and the vaccines was also commonly found in every popular social media [3, 10, 13–15]. The vaccine hesitancy among people is complicated, because it is influenced by both the context and personal factors including the national health policy, the available information, the actual and perceived vaccine efficacy and safety, perceptions about the seriousness of the epidemic, religious, social norms, health literacy, educational levels, and individual past experiences [11]. These factors may be different among countries and unique to each community of people. Understanding the vaccine hesitancy situation and relevant factors of the target population is vital for policy planning and public campaign. To date, there has been no published study of the hesitancy of Thai parents regarding COVID-19 vaccination for their children. Therefore, the purpose of this study was to examine the parental COVID-19 vaccination hesitancy (PVh) and influencing factors among Thai parents of children 5–18 years of age.

## Methods

This research was a cross-sectional survey study conducted in Thailand. The data were collected between May and June 2022 after being approved by the Human Experimentation Committee Research Institute for Health Sciences, Chiang Mai University, Chiang Mai, Thailand (No. 22/2022).

### Population and sample

The parent in this study means a father, a mother, or one who nurtures and raises a child. The inclusion criteria of the study were Thai parents of children aged between 5 and 18 years old that are eligible for the COVID-19 vaccination. [9] Parents who could not read or complete the questionnaire were excluded from the study.

The main outcome of this study was the prevalence of PVh. Based on a previous survey in Japan, 57.1% of parents expressed hesitation to vaccinate their children against COVID-19 [16]. A formula for estimating a proportion of events in a single population [17] was used with a precision level of 0.05. The sample size should not be less than 380 people. The authors decided to increase a 10% of the sample for missing or incomplete data. As a result, the sample size of this study was 420 people. The sample was selected using a convenience sampling method. [18].

### Data collection

The data were collected online using Google forms for survey. The authors distributed a QR code and a link to the questionnaire and informed consent form via online social media including Facebook and LINE applications, for institutional alumni groups, general online marketplaces and stores, and provincial news channels, where the public was members. The data collection was conducted between May and June 2022.

### Research tools

The authors developed a questionnaire based on a literature review from previous studies [3, 12, 15, 19, 20]. Constructs of the theory of planned behavior (TPB), namely, attitude towards object (the COVID-19 and the COVID-19 vaccine), attitude towards behavior (the COVID-19 vaccination program), subjective norm, and perceived behavioral control, were adopted in the questionnaire development [21]. The content validity of the draft questionnaire was assessed by three experts. They were a pediatrician and two pharmacy residents who specialized in pediatric pharmacotherapy. The item-objective congruence index (IOC) values of the questionnaire items were 0.67–1, indicating good content validity. As for the reliability test and language clarity of the draft questionnaire, it was conducted with a pilot group of 14 people. The Cronbach’s alpha coefficient was found to be 0.78. This indicated fact that the questionnaire developed was valid and reliable. The draft questionnaire and the final questionnaire were developed in Thai language. The final questionnaire consisted of two parts. The first part of the questionnaire comprised general information about the respondents and characteristics relevant to their health and experiences regarding COVID-19 and COVID-19 vaccination. The second part included 19 questions to collect opinions about parents' hesitancy to vaccinate their children against COVID-19 and five related attitude domains: four items for attitude towards COVID-19 (AC), five items for attitude towards COVID-19 vaccine (AV), four items for attitude towards COVID-19 vaccination program (AP), two items for attitude towards subjective norm (SN), and three items for parental perceived behavioral control (PC). The responses were classified into five Likert scales that were 5-extremely high or strongly agreed, 4-high or agreed, 3-moderate or neutral, 2-less or disagreed, and 1-least or strongly disagreed.

## Data analysis

Quantitative data from the survey were interpreted using descriptive statistics consisting of percentages, means, standard deviations (SD), medians, and interquartile range (IQR). The prevalence of PVh was calculated based on the PVh level at moderate or above. The Mann–Whitney *U* test for non-normally distributed data sets was used to compare the hesitancy score between groups with different experiences towards COVID-19, COVID-19 vaccination, and family context. The multiple regression analysis (MRA) was used to estimate the impact of the attitude domain on the hesitancy level. The enter technique with statistical significance at *p* value < 0.05 was applied in the MRA. All analyses were performed using SPSS version 22.0 (IBM Corp, Armonk, NY).

## Results

### General characteristics of the respondents

A total of 488 people completed the survey. Most of them (70.5%) were females. The median (IQR) age was 41 (35–47) years and 66.6% of survey respondents had a bachelor's degree or higher. They lived in different provinces across Thailand, which were primarily in Southern, Eastern, and Western regions. Altogether, 72.3% were not healthcare professionals. Over 90% of respondents were parents who hold the right to make a final decision regarding their child’s vaccination. About two-thirds of respondents were a father or a mother of the children, while the remaining were relatives of the children. About one-fourth of their children had a history of vaccine refusal, since they were extremely trpanophobic (Table 1).

**Table 1:** **G**eneral characteristics, EC&V, and FC of the respondents (*n* = 488)

Information	Yes *N* (%)
General characteristics of the respondents	
Age (years)	41 (35–47)*
Gender	
* Male*	140 (28.7)
* Female*	344 (70.5)
* Not identified*	4 (0.8)
Highest education qualification	
* Primary or lower*	35 (7.2)
* Secondary or equivalent*	128 (26.2)
* Bachelor’s or equivalent*	232 (47.5)
* Higher than bachelor’s*	93 (19.1)
Living region	
* Central*	77 (15.8)
* Northern*	136 (27.9)
* Southern, Eastern, and Western*	195 (40.0)
* Northeastern*	80 (16.4)
Relationship to children	
* Father/Mother*	330 (67.6)
* Relative*	158 (32.4)
Hold the right to make a final decision regarding their child’s vaccination	442 (90.6)
Children had a history of trypanophobia	130 (26.6)
Health care professionals	135 (27.7)
Experiences towards COVID-19 and vaccination (EC&V)	
Had ever been diagnosed with COVID-19	156 (32.0)
Family members had been diagnosed with COVID-19	201 (41.2)
The children had been diagnosed with COVID-19	134 (27.5)
Ever refused COVID-19 vaccination	66 (13.5)
Ever refused any other vaccinations	73 (15.0)
Ever refused any other vaccination for the children	62 (12.7)
Complete initial COVID-19 vaccination	472 (96.7)
Number of received COVID-19 vaccinations (shots)	3 (2–3)*
The children were living in a COVID-19 outbreak area	382 (78.3)
Family context (FC)	
Number of the children in family	2 (1–2)*
Number of family members (including the children)	4 (4–5)*
There were the children with high risk of serious COVID-19 complications due to congenital diseases	47 (9.6)
There were family members who are 60 years of age or older	460 (94.3)
There were family members with high risk of serious COVID-19 complications due to comorbidities	132 (27.0)

^*^Median (IQR)

### Experiences towards COVID-19 and vaccination (EC&V) and family context (FC)

Most of the respondents, their family members, and their children had not been diagnosed with COVID -19 (68%, 58.8%, and 72.5%, respectively). The refusal rate of COVID-19 vaccine or any other vaccines for themselves or their children were low (between 12.7% to 15%). The result showed that 96.7% of respondents had completed the initial COVID-19 vaccination with a median (IQR) of 3 (2–3) shots. Around 80% of the children were living in areas, where COVID-19 was prevalent at the time of the survey. Most of the respondents (90.4%) did not have children with a high risk of serious COVID-19 complications due to congenital diseases. However, nearly all of them (94.3%) had at least one senior family member. Besides, 27% of the respondents had family members with a high risk of serious COVID-19 complications due to comorbidities, such as diabetes mellitus, asthma, chronic obstructive pulmonary disease, cardiovascular diseases, chronic kidney disease, or immunosuppression (Table 1).

### Parental COVID-19 vaccination hesitancy (PVh) levels

The respondents who answered moderate, high, and extremely high to the question “what is your hesitancy level regarding the COVID-19 vaccination of your children?” were 32%, 16.8%, and 9.2%, respectively (Table 2). As a result, the prevalence of PVh among Thai parents in our study was 58%.

**Table 2:** Parental COVID-19 vaccination hesitancy (PVh) levels

Parental COVID-19 vaccination hesitancy (PVh) levels	*N* (%)
Least	94 (19.3)
Less	111 (22.7)
Moderate	156 (32.0)
High	82 (16.8)
Extremely high	45 (9.2)

### The comparison of PVh levels based on EC&V and FC

Parents who had previously refused to vaccinate themselves against COVID-19 and those who had previously refused to vaccinate their children against any other diseases had statistically significant higher levels of PVh than the opposite groups (3 (IQR 2.7–4) vs. 3 (IQR 2–3), and 3 (IQR 2–4) vs. 3 (IQR 2–3), respectively; *p* value < 0.001). Contrarily, the parents who had completed the initial COVID-19 vaccination had a statistically significant lower level of PVh than others (3 (IQR 2–3) vs. 4 (IQR 2–5); *p* value < 0.001). The statistically significant difference between median PVh level among parents with yes or no answer to other EC&V and FC questionnaire items were not found (Table 3).

**Table 3:** PVh levels based on EC&V and FC

Information	Median PVh level (IQR)		*p* value
	Parents with “yes” answer	Parents with “no” answer	
Experiences towards COVID-19 and vaccination (EC&V)			
Had ever been diagnosed with COVID-19	3 (2–4); * n* = 156	3 (2–3); *n* = 332	0.546
Family members had been diagnosed with COVID-19	3 (2–4); * n* = 201	3 (2–3); * n* = 287	0.937
The children had been diagnosed with COVID-19	3 (2–4); * n* = 134	3 (2–3); * n* = 354	0.220
Ever refused COVID-19 vaccination	3 (2.75–4); * n* = 66	3 (2–3); * n* = 422	0.000*
Ever refused any other vaccinations	3 (2.5–4); * n* = 73	3 (2–3); * n* = 415	0.055
Ever refused any other vaccinations for the children	3 (2–4); * n* = 62	3 (2–3); * n* = 426	0.000*
Complete initial COVID-19 vaccination	3 (2–3); * n* = 472	4 (2–5); * n* = 16	0.001*
The children were living in a COVID-19 outbreak area	3 (2–4); * n* = 382	3 (2–4); * n* = 106	0.080
Family context (FC)			
There were the children with high risk of serious COVID-19 complications due to congenital diseases	3 (2–4); * n* = 47	3 (2–4); * n* = 441	0.467
There were family members who are 60 years of age or older	3 (2–3); * n* = 460	3 (2–4); * n* = 28	0.120
There were family members with high risk of serious COVID-19 complications due to comorbidities	3 (2–4); * n* = 132	3 (2–4); * n* = 356	0.137

^*^ Mann–Whitney *U* test statistically significant difference

### Parental attitudes influencing PVh

**Fig. 1:**
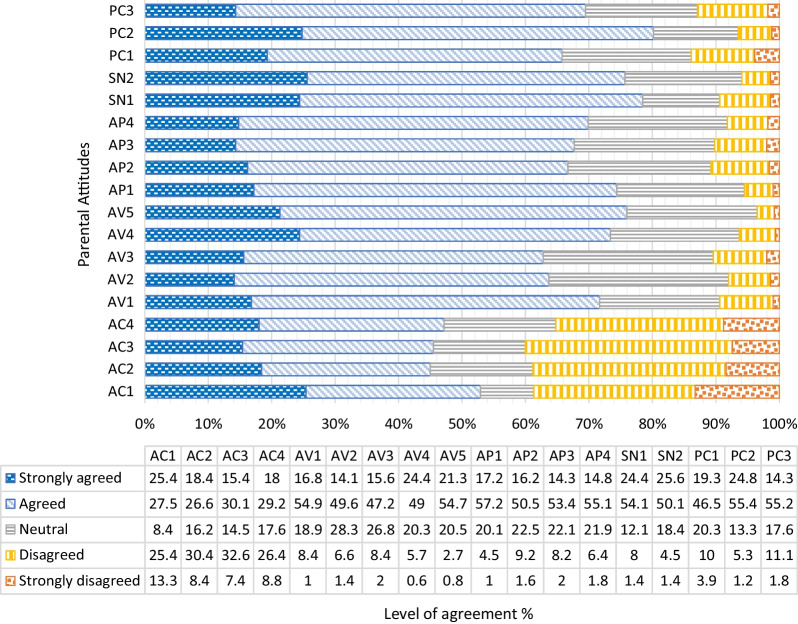
Parental attitudes towards the COVID-19, the COVID-19 vaccination program, subjective norm, and perceived behavioral control

Although the respondents had a neutral attitude towards COVID-19, they had high levels of positive attitude towards COVID-19 vaccine, attitude towards the vaccination program, parental subjective norm, and parental perceived behavioral control (Table 4 and Fig. 1). Based on the multiple regression analysis, it was found that all five domains explained PVh with their R square at 0.238. However, only attitude towards COVID-19, attitude towards COVID-19 vaccine, and parental perceived behavioral control negatively influenced PVh with statistical significance (Betas = − 0.307, − 0.123, and − 0.232, respectively) (Table 5).

**Table 4:** Parental attitudes

No	Questionnaire items	Level of agreement Mean (SD)
Attitude towards COVID-19 (AC)		
AC1	Chance of getting COVID-19 is high in children	3.26 (1.42)
AC2	Chance of complications from COVID-19, such as MIS-C or long COVID, is high in children	3.16 (1.27)
AC3	Infections with COVID-19 are more severe in children	3.13 (1.23)
AC4	Complications from COVID-19, such as MIS-C or long covid, are more severe in children	3.21 (1.26)
Attitude towards COVID-19 vaccine (AV)		
AV1	I am knowledgeable and know enough about the COVID-19 vaccine	3.78 (0.86)
AV2	The COVID-19 vaccination is effective when administered to children	3.68 (0.85)
AV3	The COVID-19 vaccine is safe when administered to children, including mine	3.66 (0.91)
AV4	Long-term safety data of the COVID-19 vaccination in children is not available	3.91 (0.85)
AV5	Potential benefits of the COVID-19 vaccination outweigh risks in my children	3.85 (0.79)
Attitude towards vaccination program (AP)		
AP1	I am satisfied with the available brand of the COVID-19 vaccine, approved for children	3.85 (0.79)
AP2	There are sufficient supplies of the COVID-19 vaccine for children with need	3.70 (0.90)
AP3	COVID-19 vaccination centers for children are sufficient and conveniently accessible	3.70 (0.89)
AP4	Time spent for receiving the COVID-19 vaccination is acceptable	3.75 (0.85)
Parental subjective norm (SN)		
SN1	Parents have a duty and responsibility to vaccinate their children	3.92 (0.90)
SN2	I wanted to fulfill my parental responsibility to live up to societal expectations	3.94 (0.86)
Parental perceived behavioral control (PC)		
PC1	To vaccinate my children is not a financial burden	3.67 (1.02)
PC2	I am certain that I can manage to vaccinate my children with the COVID-19 vaccine on time	3.97 (0.84)
PC3	I am certain that I can take care of my children if they experience any common side effects of the COVID-19 vaccine	3.69 (0.91)

**Table 5:** Multiple regression analysis of the factors influencing the PVh

Domains	b	SE	Beta	*p* value
AC: attitude towards COVID-19	− 0.323	0.042	− 0.307	< 0.001
AV: attitude towards COVID-19 vaccine	− 0.259	0.112	− 0.123	0.021
AP: attitude towards vaccination program	− 0.092	0.098	− 0.048	0.353
SN: subjective norm	− 0.006	0.084	− 0.004	0.939
PC: perceived behavioral control	− 0.388	0.095	− 0.232	< 0.001
Constant value	4.649	0.384		< 0.001

*R* = 0.487, *R*^2^ = 0.238, SEE = 1.06, *F* = 30.052, Sig. of *F* < 0.001

## Discussion

This online survey examined the parental COVID-19 vaccination hesitancy among parents of children aged 5–18 years in Thailand. Most of the respondents were parents who hold the right to make a final decision regarding their child’s vaccination. They probably had a high acceptance level of the COVID-19 vaccination, since 96.7% of them had completed the vaccine program, and the average number of the vaccines they received was around 3 shots which included the initial and booster doses.

Even though their children were living in an outbreak area and there were senior or at-risk family members, our findings revealed that 58% of Thai parents had moderate to extremely high levels of PVh. This result was in line with earlier studies conducted in other countries, such as Turkey [15], Japan [16], Italy [22] and Saudi Arabia [23–25]. The percentages of PVh in such countries had been reported as high as 52.4–72.2%. Issues regarding confidence in the vaccine efficacy and safety, quality uncertainty, and lack of adequate available information were cited as the contributing factors to the high level of PVh in those studies [15, 16, 22, 23]. Although some recent studies conducted in the United States [26, 27], Malaysia [28], and South Korea [29] found that PVh prevalences were considerably lower than our finding (15–28.9%), the above contributing factors of PVh were still indicated [26–28].

Previous refusal to receive the COVID-19 vaccine for themselves and completing the initial COVID-19 vaccination were associated with higher and lower PVh, respectively. Those results indicated that the direct experience of the parents with their COVID-19 vaccination was one of the key factors influencing PVh. This hypothesis was supported by previous studies which found that there was an inverse relationship between COVID-19 vaccination history of the parents and PVh [15, 16, 28, 30]. Therefore, a campaign to create a positive attitude towards vaccination for themselves and increase the rate of COVID-19 vaccination among parents, in addition to the promotion of their child’s vaccination should be conducted.

Our study also found that PVh was higher with statistical significance among parents who previously refused any other vaccinations for their children. These parents may have misunderstandings, distrust, excessive fear, and concerns about any vaccination, especially COVID-19 vaccines which had been manufactured by newly invented platforms for an unfamiliar emerging disease [31]. As a result, a history of incomplete vaccination for other diseases of the children may be a screening tool for this group of parents [32]. Special consultation with emphasis on the seriousness of COVID-19 problems in their children and the positive facts and information with proper media should be applied [33–35].

In previous studies, parents who had a family member who suffered or died from the disease showed a lower level of PVh [30]. Although those devastating experiences can increase the perceived threat of the disease, the perceived benefits and risks of the vaccine may not be changed. Unsurprisingly, our study did not find a statistical difference in PVh between parents who had or did not have direct experiences with COVID-19. Thus, measures to increase the perceived benefits and decrease the perceived risk of the vaccine should be considered.

Parental subjective norm (SN), perceived behavioral control (PC2), and attitude towards vaccine regarding the unavailability of long-term safety (AV4) were rated with high levels of agreement in our study. However, the multiple regression analysis found that only attitude towards COVID-19, attitude towards COVID-19 vaccine, and parental perceived behavioral control statistically influenced PVh with negative beta values indicating the inverse relationship between those factors and the level of hesitancy. As a result, communication to increase the perceived risk of COVID-19, the perceived benefit of COVID-19 vaccine, and the perceived behavioral control could be the most effective directions to reduce the level of parental COVID-19 hesitancy [36, 37].

To our knowledge, this is the first study to explore the parental COVID-19 vaccine hesitancy in Thailand. Most of the respondents were parents who hold the right to make a final decision regarding their child’s vaccination. The questionnaire was systematically developed in Thai language and tested for its validity and reliability. Therefore, their opinions collected in our study could be highly correlated with the actual decision for their children in the near future.

Although our study was conducted in various living regions which improved the generalizability of our results, some limitations require consideration. First, the study was an online survey. This could be of concern that only parents who were familiar with an online questionnaire and well-equipped can participate in the data collection. Thanks to several national projects of the Thai government such as Thai-Cha-Na (mobile application for tracking COVID-19 contact persons) and Mor-Prompt (mobile application for COVID-19 vaccine services) which most Thai people used in everyday life, nowadays, Thai parents could participate in the online survey without any limitations as aforementioned. Secondly, we conducted this study during a period when the incidence of severe COVID-19 was relatively low. The parental vaccine hesitancy was sensitive to the context of data collection, e.g., outbreak situation and trend, news, rumors on public and social media, national and local policy, as well as local availability of the vaccine. The prevalence of PVh in this study was calculated based on the PVh level at moderate or above. Different cutoff PVh levels for data transformation, such as determining only high and extremely high PVh levels could lead to remarkably different prevalence [32]. Therefore, it is important to use caution when applying the study's findings to other contexts. Further prospective multi-centered studies should be conducted in a larger population to increase the generalizability and address the effective measures to overcome the COVID-19 vaccination hesitancy.

## Conclusions

The parental COVID-19 vaccination hesitancy among Thai parents of children aged 5–18 years was prevalent. The hesitancy level was higher among parents who refused their COVID-19 vaccination or denied any other vaccinations for their children. Contrarily, parents who had completed the initial COVID-19 vaccination had lower vaccine hesitancy. Past experiences regarding the parents and children vaccination could be considered as a screening tool for the risk of vaccine hesitancy. Factors influencing the hesitancy of Thai parents were multifactorial, especially attitudes towards COVID-19, attitudes towards COVID-19 vaccine, and perceived behavioral control. Parents and public education should emphasize on threats and consequences of COVID-19 and the risk–benefit ratio of COVID-19 vaccine as well as inspire the confidence of the parents regarding their child’s vaccination.

## Data Availability

The data sets used and/or analysed during the current study are available from the corresponding author on reasonable request.

## Abbreviations

AC: Attitude towards COVID-19
AP: Attitude towards COVID-19 vaccination program
AV: Attitude towards COVID-19 vaccine
COVID-19: The coronavirus disease 2019
EC&V: Experiences towards COVID-19 and vaccination
FC: Family context
IOC: Item-objective congruence index
IQR: Interquartile range
MRA: Multiple regression analysis
PC: Perceived behavioral control
PVh: Parental COVID-19 vaccination hesitancy
SD: Standard deviation
SN: Attitude towards subjective norm
TPB: The theory of planned behavior
WHO: The World Health Organization
